# Mapping pachytene chromosomes of coffee using a modified protocol for fluorescence *in situ* hybridization

**DOI:** 10.1093/aobpla/plt040

**Published:** 2013-09-05

**Authors:** Ana Amélia Sanchez Iacia, Cecília A. F. Pinto-Maglio

**Affiliations:** Centro de Pesquisa e Desenvolvimento de Recursos Genéticos Vegetais, Instituto Agronômico de Campinas, CxP. 028, Campinas, São Paulo 13012-970, Brazil

**Keywords:** *Coffea*, FISH, meiotic chromosomes, molecular cytogenetics, physical mapping, rDNA.

## Abstract

Fluorescence *in situ* hybridization (FISH) is the most direct method for physically mapping DNA sequences on chromosomes. Fluorescence *in situ* hybridization mapping of meiotic chromosomes during the pachytene stage is an important tool in plant cytogenetics, because it provides high-resolution measurements of physical distances. Fluorescence *in situ* hybridization mapping of coffee pachytene chromosomes offers significant advantages compared with FISH mapping of somatic chromosomes, because pachytene chromosomes are 30 times longer and provide additional cytological markers. However, the application of this technique to pachytene chromosomes has been complicated by problems in making preparations of meiotic chromosomes and by difficulties in the application of standard FISH protocols. We have been able to overcome most of these obstacles in applying the FISH technique to the pachytene chromosomes of coffee plants. Digesting the external callose layer surrounding the pollen mother cells (PMCs) in conjunction with other procedures permitted suitable pachytene chromosomes to be obtained by increasing cell permeability, which allowed the probe sequences to enter the cells. For the first time, hybridization signals were registered on coffee pachytene chromosomes using the FISH technique with a repetitive sequence as a probe. We obtained slides on which 80 % of the PMCs had hybridization signals, resulting in FISH labelling with high efficiency. The procedure does not seem to be dependent on the genotype, because hybridization signals were detected in genetically different coffee plants. These findings enhance the possibilities for high-resolution physical mapping of coffee chromosomes.

## Abstract (precis)

FISH-mapping to meiotic chromosomes at pachytene is an important tool in plant cytogenetic research as it provides good resolution measurements of physical distances. This publication brings a new and more efficient protocol for the application of FISH technique for the first time in meiotic pachytene chromosomes of coffee.This new protocol involves some procedures for obtain suitable pachytene chromosomes that allows the making of a higher-resolution cytogenetic mapping on coffee chromosomes than that mapping on mitotic chromosomes. The use of this method expands the possibilities for high definition physical mapping of coffee chromosomes.

## Introduction

Genetic physical and linkage maps are highly useful tools that constitute ‘road maps’ with marks and guide signs designed for both genetic work and selection. Physical maps are the chromosomes fully cloned in large and aligned DNA fragments such as bacterial artificial chromosomes (BACs). Genes of relevant biological or agronomic importance can be physically located and eventually modified as desired. Linkage maps are made by genetic linkage studies and the distances are measured in arbitrary Morgan units. Thus, the distances between markers are estimated and lack precision when compared with the number of bases, as in the case of physical maps. Both maps can be integrated and the linkage marks fully anchored in the physical genome. The availability of physical maps eases the process of gene location and of sequencing by traditional methods.

Although linkage maps are reasonably good at describing the order of genes and the amount of crossing over between them, they do not indicate the actual physical location of genes on chromosomes. This is because the frequency of crossing over along the chromosome length is not uniform, and loci that are physically distant on chromosomes can appear close together in linkage maps, and vice versa (Ganal *et al.* 1989; Zhong *et al.* 1996; Islam-Faridi *et al.* 2002). These discrepancies hinder the isolated use of linkage maps to align the sequences of a genome or even to discover the existence of transposable elements (Budiman *et al.* 2004). Eventually, distances between DNA markers need to be described not only by recombination frequency, but also by actual physical distance.

Fluorescence *in situ* hybridization (FISH) is a cytogenetic technique developed to detect and localize specific DNA sequences on chromosomes. It uses fluorescent probes that bind only to targets when there is a high degree of sequence complementarity (Jiming and Gill 2006; Figueroa and Bass 2010). Using FISH with specific locus probes is a relatively fast way to gain access to the position of the corresponding DNA sequences on intact chromosomes. Through this technique, it is possible to correlate molecular markers with specific chromosomes and integrate physical and linkage maps (Figueroa and Bass 2012; Lou *et al.* 2013). The resolution of the physical mapping based on FISH depends on the degree that chromosomes are condensed. Meiotic chromosomes at the pachytene stage are better than the mitotic chromosomes for high-resolution physical mapping because they are less condensed than their somatic counterparts. The distended chromatin condition of the pachytene chromosomes enhances FISH resolution. This resolution could reach 100 KBs being possible to distinguish two or more small-sized sequences even when positioned very close (Peng *et al*. 2012).

In *Oryza*, FISH mapping of pachytene chromosomes resulted in the construction of a high-resolution map that allowed the determination of sequences at a resolution of ∼40 kb. However, for mapping chromosomes in mitosis in the same material, the minimum resolution required to distinguish the sequences was 2 Mb (Cheng *et al.* 2002).

Fluorescence *in situ* hybridization mapping of meiotic chromosomes at the pachytene stage has been developed for *Brassica* (Armstrong *et al.* 1999), *Medicago* (Kulikova *et al.* 2001), *Arabidopsis* (Ziolkowski and Sadowski 2002), *Zea* (Amarillo and Bass 2007; Figueroa and Bass 2012) and *Camellia* (Vijayan *et al.* 2012).

The pachytene chromosomes of tomato (*Solanum lycopersicum*) have been widely mapped by the FISH technique. The pachytene chromosome 2 of tomato was mapped with the rDNA 45S (pTa71) sequence, BACs and a telomere sequence from the *Arabidopsis* genome (pAtT4) and this physical map provided extensive coverage of the heterochromatic regions, mainly at the limit to the euchromatic region. This high-definition map complemented that of heterochromatic regions, where genetic mapping was impracticable due to the unequal distribution of recombination points along this chromosome (Koo *et al.* 2008).

The genus *Coffea* comprises predominantly diploid species with 2*n* = 2*x* = 22 chromosomes that are mostly self-incompatible, but with a few self-compatible diploid species such as *C. heterocalix* and *C.* sp. *Moloundou*. The only polyploid and self-compatible species recorded to date is *C. arabica*.

The most commercially important species of coffee are *C. arabica* and *C. canephora*. Hybrids between these two species with 2*n*= 4*x* = 44 chromosomes are called ‘arabusta’ and were created for breeding purposes. This hybrid has been obtained in various ways. The arabusta material we used in this study was derived from hybrids obtained by Mendes and Bacchi (1940) and Mendes (1944). One of the parents of this hybrid, *C. canephora* cv. Robusta, was obtained by doubling the number of chromosomes in a normal diploid (2*n* = 22), using colchicine treatment. The other parent, *C. arabica* cv. Bourbon Vermelho, was derived from a dihaploid (*n* = 22) in which the chromosomes were doubled by colchicine treatment. By crossing these plants, these authors obtained the F_1_ arabusta hybrid. After this, several F_2_ arabusta plants were created by self-pollination of the F_1_ arabusta hybrid and backcrosses to the arabica parent.

Several cultivated and wild species of coffee have been characterized by their mitotic chromosomes with FISH mapping using repetitive sequences of 45S rDNA and 5S regions (45S–pTa71) (5S–pSct7) and BACs linked to resistance genes as probes (Lombello and Pinto-Maglio 2004*a*, *b*, *c*; Pinto-Maglio 2006; Herrera *et al.* 2007; Hamon *et al.* 2009). However, coffee mitotic chromosomes in metaphase are very small (1–3 µm) and have similar morphologies, making their individual identification difficult (Krug 1934; Krug *et al.* 1939; Mendes 1957). These characteristics have prevented the construction of a molecular cytogenetic map and the saturation of a physical map for each chromosome.

Pachytene chromosomes of coffee plants are 30 times longer than their somatic counterparts at metaphase (Pinto-Maglio and Cruz 1987, 1998). They also provide additional cytological markers, including a clear distribution pattern of distal euchromatin and proximal heterochromatin segments. These features have allowed the construction of a pachytene karyotype and an ideogram for *C. arabica* (Pinto-Maglio and Cruz 1998). This characterization of the meiotic chromosomes of *C. arabica* opened up the possibility of applying the FISH mapping technique to coffee pachytene chromosomes, similar to that achieved with *Sorghum* (Islam-Faridi *et al.* 2002; Kim *et al.* 2005), *Solanum* (Van der Knaap *et al.* 2004; Koo *et al.* 2008; Tang *et al.* 2009; Lou *et al.* 2010), *Daucus* (Iovene *et al.* 2011) and *Gossypium* (Ji *et al.* 2007; Peng *et al.* 2012).

The pachytene chromosomes of coffee are morphologically similar to tomato pachytene chromosomes. Both species have chromosomes with similar morphology that allows individualized identification of each chromosome into the chromosome complement (Pinto-Maglio and Cruz 1998; Lin *et al.* 2005).

In our laboratory, numerous attempts to apply FISH mapping to coffee pachytene chromosomes, even using probes composed of repetitive sequences such as rDNA 45S and 5S, resulted in few slides with hybridization signals.

Unfortunately, the same FISH protocol that was established for somatic coffee chromosomes (Lombello and Pinto-Maglio 2004*a*, *b*, *c*; Pinto-Maglio 2006) did not work when it was applied to the coffee microspore mother cells, and positive hybridization signals were rarely observed on pachytene chromosomes in our experiments.

In flowering plants, callose is synthesized and located around the microspores during the development of the male gametophyte, where it remains until pollen wall formation. The callose may function to protect the novel male gametophytes from hostile interactions with the parental genotype and perhaps to individualize the spores within the tetrad. In the absence of callose, the microspores do not separate, and the pollen grains become sterile (Enns *et al.* 2005; Huang *et al.* 2009). The typical callose layer that surrounds the pollen mother cells (PMCs) of coffee during meiotic division could be a barrier to the entry of the probe sequences in these cells.

We hypothesized that if FISH probe hybridization to DNA targets on chromosomes is indeed prevented by the presence of callose then hydrolysis of the callose layer that surrounds the PMCs might increase cell permeability, allowing the entry of probe sequences and, consequently, hybridization.

In this study, we describe an effective technique combining the preparation of pachytene chromosomes of coffee with a modified FISH protocol that allows a high-resolution physical mapping.

## Methods

### Plant material

Floral buds in the early stages of development (around 5 mm in length) were collected 30–15 days before anthesis, from 17 hybrid coffee plants of arabusta F_2_ (*C. arabica* cv. Bourbon Vermelho × *C. canephora* cv. Robusta). The plants used in this study were F_2_ arabusta hybrids that were obtained by selfing of an F_1_ arabusta hybrid and backcrosses to the arabica parent. These plants exhibit different morphologies and meiotic studies have shown that they have different meiotic behaviour with various degrees of abnormalities and fertility (Granato 2010).

Samples of about 100 floral buds per plant were fixed in Carnoy’s solution (ethanol : glacial acetic acid 3 : 1) and then degassed with a vacuum pump for 5 min. The buds were stored for 24 h at room temperature and then at −20 °C.

### Chromosome preparation

A squash technique was used for the cytological preparations and about 200 slides were prepared for each plant. The anthers were removed from the buds and washed twice for 5 min in a cooled sodium citrate buffer (0.1 M sodium citrate, 0.1 M citric acid, pH 4.5).

To test our hypothesis that removal of the callose barrier around the PMCs might facilitate FISH reagent uptake, we subjected the anthers to hydrolysis in a mixture of the enzymes: cellulase (Sigma C-1794), cytohelicase (Sigma C-8274), pectolyase (Sigma P-5936) and β-(1→3)-d-glucanase (Sigma-Fluka 67138-10MG) for various time intervals (see Table 1 for protocols). After all enzyme hydrolysis treatments, the anthers were then placed in 60 % acetic acid for 30 min as an additional hydrolysis step. Following hydrolysis, the chromosomes were prepared by macerating two anthers per slide in 60 % acetic acid, subsequent removal of all visible debris. The slides, with only PMCs, then had a coverslip coated with liquid silicone (dimethyldichlorosilane; Sigma D-3879) applied, and after heating briefly over a spirit flame they were pressed to promote cell spreading. The coverslips were removed after freezing with liquid nitrogen, and the slides were dehydrated in an ethanol series (2 min each in 70, 90 and 100 %) and allowed to dry at room temperature. Using a phase-contrast microscope, the best slides with the following characteristics were selected: around 700 PMCs present (see below), most of which showed no callose, and with the PMCs thoroughly squashed but without their contents being dispersed. Only the slides with these characteristics were subjected to FISH.

**Table 1.** Hydrolysis tests of arabusta coffee anthers with different concentrations and combinations of enzymes and hydrolysis times.

Test	Enzyme concentration	Hydrolysis time (h)
A	0.2 % cellulose, 0.2 % cytohelicase, 0.2 % pectolyase	4
B	0.2 % cellulase, 0.2 % cytohelicase, 0.2 % pectolyase, 0.05 % glucanase	2, 3, 4, 5, 6
C	0.3 % cellulase, 0.3 % cytohelicase, 0.3 % pectolyase, 0.05 % glucanase	3, 4

### DNA probe

The probe that was used for the FISH reactions was a 45S rDNA sequence (26S, 5.8S and 18S) from the wheat genome (*Triticum aestivum*) that was cloned into a pUC19 plasmid (9 kb; pTa71) (Gerlack and Bedbrook 1979). The labelling was performed with digoxigenin-11-dUTP by the nick translation reaction, according to the manufacturer’s instructions (Roche, Berlin, Germany).

### FISH protocol

The FISH protocol was based on that used for somatic coffee chromosomes (Pinto-Maglio *et al.* 2001). Slides were pretreated with 100 mg ml^–1^ DNase-free RNase in a moist chamber for 2 h at 37 °C and then washed three times for 5 min in 2× saline-sodium citrate (SSC) buffer (two washes at room temperature and one at 37 °C). The slides were incubated in 0.05 % pepsin (Sigma P-7125) with 10 mM HCl for 10 min at 37 °C to permeabilize the PMCs. Finally, the slides were washed twice for 5 min in 1× phosphate-buffered saline (PBS), once for 10 min in 2.5 % formamide and once for 5 min in 1× PBS. After these washes, the slides were dehydrated in an ice-cold ethanol series (70, 90 and 100 %, 2 min for each ethanol concentration) and dried at room temperature.

### Chromosome and rDNA probe denaturation

Chromosome denaturation was performed by heating the slides on a hot plate at 80 °C for 2 min with 50 µl of a denaturation mixture (70 % formamide, 50 mM sodium phosphate, pH 7, 2× SSC, H_2_O). They were then immediately immersed in an ethanol series (70, 90 and 100 %, 2 min for each ethanol concentration) at −20 °C. The probe was labelled with digoxigenin, diluted (10 ng µl^–1^) in a hybridization mixture (50 % formamide, 2× SSC, 50 mM sodium phosphate, pH 7, 10 % dextran sulfate) and then denatured at 99 °C for 8 min.

### Hybridization and post-hybridization washes

For the hybridization, 15 µl of the probe were added to the slides, which were then incubated in a moist chamber for 18 h at 37 °C. The slides were then successively washed six times for 5 min in 1× SSC (three times at 37 °C and three times at 60 °C), three times for 5 min in TNT buffer (0.1 M Tris–HCl, pH 7.5, 0.15 M NaCl, 0.5 % Tween 20) and once for 5 min in 4T buffer (4× SSC, 0.5 % Tween 20). The slides were incubated in 4B buffer (4× SSC, 0.5 % blocking reagent—Roche Nucleic Detection Kit-11 175 041 910) for 20 min at 37 °C, followed by three 10-s washes in 4T buffer. Finally, the slides were dried at room temperature.

### Detecting signals and images

To detect the probe hybridization sites, 50 μl (5 ng µl^–1^) of anti-digoxigenin/fluorescein isothiocyanate (FITC) were added to each slide, and the slides were incubated in a moist chamber at 37 °C for 45 min. The slides were washed twice for 5 min in TNT buffer with gentle agitation. After these washes, the slides were dehydrated in an ethanol series (70, 90 and 100 %, 2 min for each ethanol concentration) and dried in the dark at room temperature. Coverslips were applied using an antifade mounting medium (Vectashield^®^, Vector) containing 4′,6-diamidino-2-phenylindole (DAPI). The slides were analysed using an Olympus BX50 fluorescence microscope that was equipped with an Olympus CCD (charge-coupled device) digital chamber Q-Color with refrigeration. The images were processed with Image-Pro Plus software, version 6.0 (Media Cybernetics).

## Results

The application of the hydrolysis protocols listed in Table 1 resulted in two basic types of cytological preparations: (i) slides with mostly intact PMCs with well-spread cytoplasm and no callose layer and (ii) slides with similar PMCs but in which the callose was only partially hydrolysed, i.e. some callose and residual digested material (debris) remained.

The average number of PMCs present on each slide was estimated using the area of a coverslip (24 × 24 mm), which, with uniform coverage, indicated the presence of ∼630 PMCs per slide. Uniform preparations with this average number of PMCs per slide were ensured by means of: (i) increased adhesion of cells on the slides that were subjected to cleaning with 6 N hydrochloric acid (Schwarzacher and Heslop-Harrison 2001) and (ii) decreased detachment of PMCs from coverslips with the use of silicone (dimethyldichlorosilane, Sigma) which prevents the loss of material when the coverslip is detached following freezing with liquid nitrogen.

**Figure 1.**
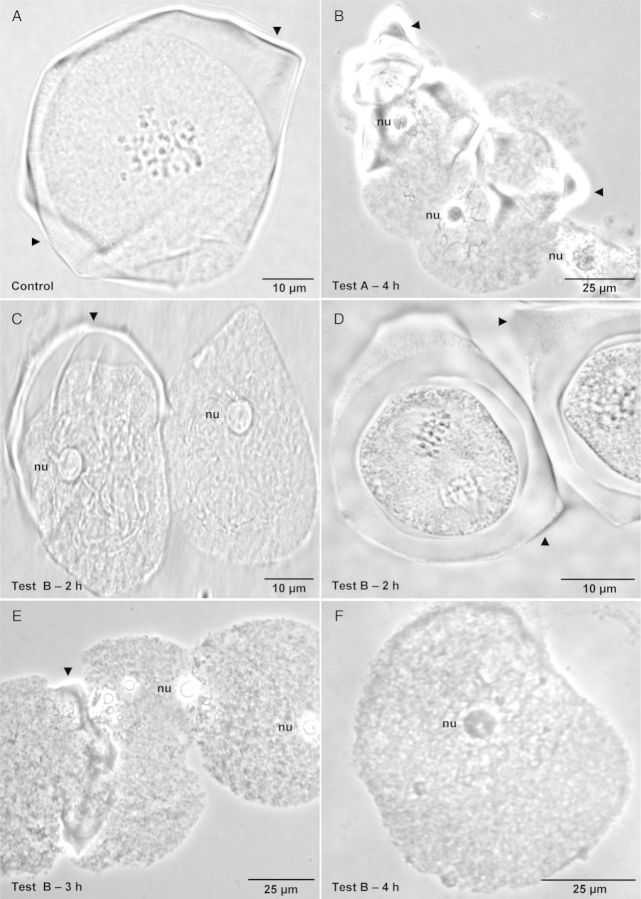
Pollen mother cells of coffee after different hydrolysis tests for callose layer removal. (A) Metaphase I—control without hydrolysis; (B) prophase I—Test A—4 h without glucanase; (C) and (D) prophase I and metaphase II—Test B—2 h with glucanase; (E) prophase I—Test B—3 h with glucanase; (F) prophase I—Test B—4 h with glucanase. The images were acquired by phase-contrast microscopy. nu, nucleolus. Callose is indicated by an arrowhead.

Depending on the extent of callose dissolution by the combination of enzyme mixtures and incubation times (tests A, B or C), we obtained percentages of PMCs with hybridization signals of 20–80 % per slide (Table 2). Control slides that were not subjected to any enzymatic treatment revealed PMCs with a normal layer of callose that was thin in the early stages of meiosis and progressively thickened in the final stages (Fig. 1A). In these slides, no hybridization signals were observed at the nucleolar organizing regions (NORs), only background signals.

**Table 2.** Results of the hybridization signals obtained by FISH using the pTa71 probe after hydrolysis tests of the anthers of pollen mother cells of arabusta coffee hybrids.

Test	Enzyme concentration	Hydrolysis time (h)	Callose in PMCs	PMCs with FISH signals per slide (%)
A	0.2 % cellulase, 0.2 % cytohelicase, 0.2 % pectolyase	4	++++	20
B	0.2 % cellulase, 0.2 % cytohelicase, 0.2 % pectolyase, 0.05 % glucanase	2	+++	30
		3	++	50
		4	–	80
C	0.3 % cellulase, 0.3 % cytohelicase, 0.3 % pectolyase, 0.05 % glucanase	3	+	50
		4	–	60

### Test A

The slides prepared with anthers that were hydrolysed with cellulase, cytohelicase and pectolyase but without glucanase for 4 h exhibited PMCs with some remnants of callose that varied in thickness (Fig. 1B). This test resulted in slides with the greatest number of PMCs containing callose and debris.

**Figure 2.**
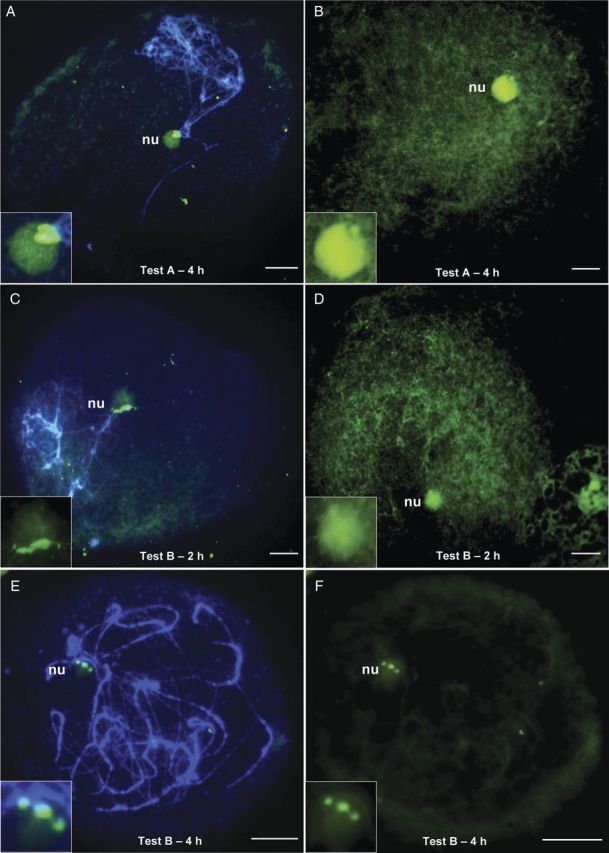
Fluorescence *in situ* hybridization mapping of the rDNA 45S sequence (pTa71) in prophase I chromosomes after different hydrolysis tests on coffee PMCs for callose layer removal. The probe was labelled with digoxigenin/FITC, and the chromosomes were counterstained with DAPI. The tests resulted in different percentages of PMCs with hybridization signals at NORs (in merged images on the left and in F) and in PMCs without signals, on the right, images with FITC only. (A) and (B) Test A—4 h without glucanase. (C) and (D) Test B—2 h with glucanase. (E) and (F) Test B—4 h—same PMC with signal. Scale bars, 10 µm.

After FISH, these slides showed only 20 % of PMCs with NOR hybridization signals (Table 2). NOR signals were observed only in PMCs with clear cytoplasm (Fig. 2A), and PMCs with dense cytoplasm (as shown in Fig. 2B) resulted in no hybridization.

### Test B

These slides were prepared with anthers that were subjected to hydrolysis with a mixture of the former three enzymes with the addition of glucanase for periods of 2, 3, 4, 5 and 6 h. Hydrolysis for 2 h resulted in slides with some PMCs containing callose and others without callose. The callose layer was thicker and consistent in PMCs in later phases of meiosis (Fig. 1C and D). After FISH, 30 % of these PMCs showed bivalents with signals in the NORs (Table 2); some of them had clear signals (Fig. 2C) while others had no signal, especially those with a dense cytoplasm (Fig. 2D). Hydrolysis for 3 h resulted in slides containing a majority of PMCs without callose, although some cells showed remnants (Fig. 1E). After FISH, these slides showed 50 % of PMCs with chromosomes labelled with hybridization signals in the NORs (Table 2). Hydrolysis with Test B–for 4 h resulted in the best slides, as they showed no callose in the PMCs, and they were free of debris. The cells also presented a clear cytoplasm with good integrity and spreading (Fig. 1F). After FISH, these slides showed ∼80 % of PMCs with chromosomes containing hybridization at NORs with no background, which allowed a clear visualization of hybridization signals (Table 2, Fig. 2E and F).

A reduced number of PMCs, even some that lacked the callose layer, showed no hybridization signals in the bivalents. However, these PMCs were located near the edges of the coverslips and this may have negatively influenced the FISH result.

Test B slides that were incubated for 5 and 6 h resulted in PMCs with aspects of excessive hydrolysis, i.e. disintegration. This condition prevented the proper handling of cells during the slide preparation, making it impossible to remove debris. Due to these characteristics, these slides were not subjected to FISH.

### Test C

To test the activities of the enzymes cellulase, cytohelicase and pectolyase, we used Test C, in which the concentration of these three enzymes was increased from 0.2 to 0.3 % while the concentration of glucanase was maintained at 0.05 %. Hydrolysis with Test C–3 h resulted in slides containing PMCs with few remnants of callose and after FISH, 50 % of PMCs showed hybridization signals on NORs (Table 2). However, despite these results, the slides still presented a background signal. Hydrolysis with Test C—4 h—resulted in slides with no callose surrounding the PMCs. Fluorescence *in situ* hybridization resulted in 60 % of the slides containing PMCs with hybridization signals, and there was no background signal.

**Figure 3.**
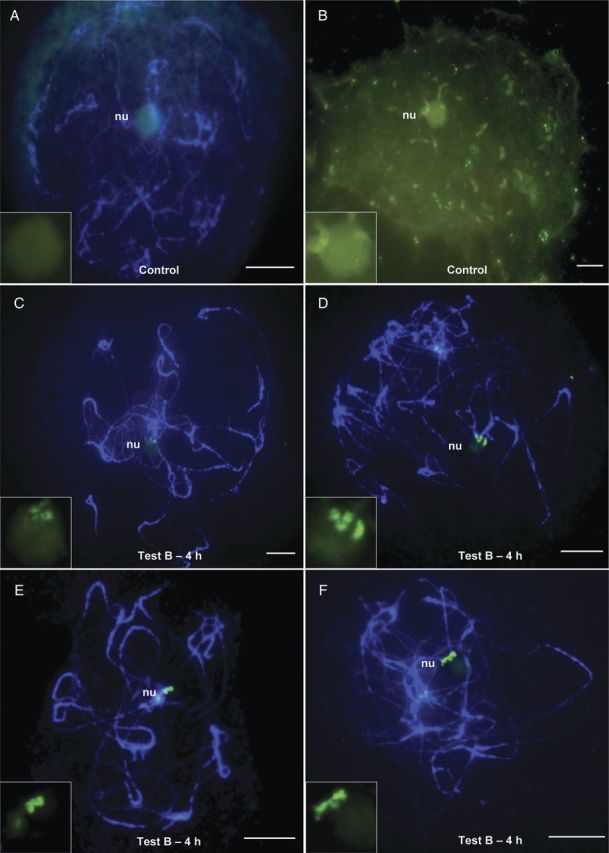
Differences in FISH mapping of rDNA 45S sequence (pTa71) in prophase I chromosomes of PMCs of coffee that were subjected to control conditions and hydrolysis Test B—4 h for callose layer removal. (A) and (B) Control PMCs without hydrolysis show no hybridization signals at the NORs (on the left in the merged images; on the right only in FITC); (C)–(F) PMCs with bivalents at the pachytene stage with hybridization signals at the NORs after hydrolysis with Test B—4 h containing glucanase. Bars = 10 µm.

The PMCs of the control slides showed a thick cytoplasm and total absence of hybridization signal (Fig. 3A and B), while the PMCs that were hydrolysed with Test B–4 h showed clean cytoplasm and strong hybridization signals (Fig. 3C–F). The signals regarding the NOR sites in Fig. 3C–F vary in number due to the pairing of bivalents.

The hydrolysis tests applied in the present study, with and without glucanase, were less effective in slides containing PMCs in meiotic stages beyond metaphase I since in these the hybridization signals in the PMCs were absent.

This lack of signal was associated with the presence of a non-reduced callose layer in PMCs, and this correlates with our observations that from metaphase I until the final stages of meiosis, the callose layer becomes progressively thicker until the pollen grain is formed.

**Figure 4.**
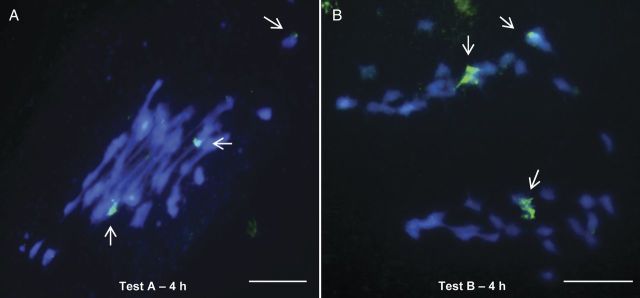
Fluorescence *in situ* hybridization mapping of rDNA 45S (pTa71) in coffee PMCs. Digoxigenin-labelled probe/FITC and chromosomes counterstained with DAPI. Segregating bivalents with hybridization signals at (A) metaphase I after hydrolysis with Test A—4 h without glucanase; (B) anaphase I after hydrolysis with Test B—4 h containing glucanase. Hybridization signals in PMCs were recorded in segregating homologues in metaphase I and anaphase I only in those cells for which the callose was mechanically disrupted by squashing. Hybridization signals are indicated by arrows. Bars = 10 µm.

Probably as a consequence of this thickened callose layer, glucanase, or at least glucanase at the concentration used in the present study, was ineffective from metaphase I phase onwards, as shown in Fig. 1D, which demonstrates that the PMCs in metaphase II have an intact layer of callose, even after hydrolysis with Test B for 2 h. Hybridization signals in PMCs were recorded in segregating homologues in metaphase I, and anaphase I but only in those cells for which the callose was mechanically disrupted by squashing (Fig. 4).

The additional hydrolysis with 60 % acetic acid was used for slides with coffee PMCs, since this treatment reduced the amount of debris and the background signal.

Independent of the hydrolysis conditions, when slides that were subjected to 16 and 18 h of hybridization were compared, differences in the quantity and quality of the FISH signals obtained were observed. The slides that were hybridized for 18 h displayed a greater number of PMCs with a better quality of signals than slides that were hybridized for 16 h.

The probe was labelled by direct and indirect labelling. These different methods led to different FISH results in coffee PMCs. In cytological preparations in which the probe (pTa71) was labelled for indirect detection, i.e. by an immunochemical reaction, the hybridization sites were more clearly defined and yielded stronger signals.

In contrast, in preparations in which the labelled probe was used for direct detection, the presence of background signal was detected in the cytoplasm of PMCs, impairing the detection of positive hybridization signals. In both cases, the result was independent of the hydrolysis method.

The procedures in the present study were applied to 17 hybrid coffee plants of arabusta F_2_, and we obtained uniform results for all specimens in both the quality and quantity of signals obtained for PMCs.

## Discussion

In a cytogenetic map, genes and DNA sequences can be localized on their respective chromosomes and are physically associated with specific signals by classical cytogenetic techniques, including C-banding, chromomycin and DAPI banding, or cytomolecular techniques, particularly the FISH technique.

Since 1980, the use of the FISH technique associated with other technological advances has become common in the genomic characterization of plant species by localizing different types of DNA sequences and has been included in the elaboration of physical maps using libraries of BACs (Wang *et al*. 2006; Tang *et al*. 2009).

Fluorescence *in situ* hybridization mapping of mitotic chromosomes in metaphase has contributed to the expansion of knowledge of the functional genomes of various species. However, this type of FISH mapping in somatic chromosomes results in low resolution maps. For small chromosomes, the low resolution makes it difficult to localize the sites of hybridization, particularly for short sequences and/or proximal sequences, i.e. sequences localized near the centromere.

The application of FISH to coffee mitotic chromosomes has provided new opportunities for identifying chromosomes and mapping genes and sequences of interest in several species, but coffee somatic chromosomes are small and have similar morphologies, so the exact location of a small repetitive sequence in this type of chromosome is almost impossible to determine.

Contrary to what occurs with the somatic chromosomes, the meiotic chromosomes of coffee (*C. arabica*) at the pachytene stage of prophase I are approximately 30 times greater in length than their corresponding somatic chromosomes. In addition to their longer length, these bivalents have well-defined characteristics such as heterochromatin distribution, chromomeres and the locations of NORs and centromeres.

A pachytene karyotype is avaliable for *C. arabica* as well as a karyogram and an ideogram (Pinto-Maglio and Cruz 1998). This fact makes it possible to recognize the 22 bivalents of this species and therefore allows them to be mapped by high-resolution FISH.

In our laboratory, meiotic preparations of coffee PMCs in the pachytene stage have been subjected to the same FISH protocol that has been applied to mitotic coffee chromosomes, but unfortunately the hybridization signals were not routinely observed. When these signals are present, they do not appear uniformly on all PMCs in the slides. One difference between meristematic cells (somatic chromosomes) and PMCs (meiotic chromosomes) is a thick layer of callose that is characteristic of the latter.

We hypothesized that the callose surrounding the PMCs acted as a barrier that prevents successful use of FISH reagents. Callose is a polysaccharide in the form of β-1,3-glucan, which is degraded by β-1,3-glucanase (Chen and Kim 2009), and so this enzyme is an obvious choice in any hydrolysis treatment.

However, other workers have used the enzymes cellulase, cytohelicase and pectolyase, but without glucanase, to successfully apply the FISH technique to pachytene chromosomes in alfalfa (Kulikova *et al.* 2001) and tomato (Zhong *et al.* 1996).

We therefore used various combinations and concentrations of these four enzymes in our attempts to overcome the callose barrier in coffee, and we experimented with varying hydrolysis times with these enzymes (Table 1); and since other protocols for FISH mapping of pachytene chromosomes include a further hydrolysis step with 60 % acetic acid (Ziolkowski and Sadowski (2002) with *Brassica*, Capdeville *et al.* (2009) with banana, and Peng *et al.* (2012) with cotton) we also included this secondary treatment. On coffee this step resulted in suitable slides. Other authors reported that the cleaning action of acetic acid on the slide increases the mobility of the probe (de Jong *et al.* 1999; Adeleke *et al.* 2002).

Of the various procedures we adopted, that using a mixture of 0.2 % cellulase, 0.2 % cytohelicase, 0.2 % pectolyase and 0.05 % glucanase for a hydrolysis time of 4 h gave PMCs with no callose and clear cytoplasm, and after FISH showed ∼80 % of PMCs with chromosomes containing hybridization at NORs with no background, and allowed a clear visualization of hybridization signals (Table 2). Thus, the hydrolysis tests influence the FISH results, and adjustments in the methodology of this technique have also contributed to improvements. For example, the application of different hybridization periods has resulted in differences in the quantity and quality of the FISH signals obtained.

We observed that the 16 and 18 h of hybridization period showed differences in the quantity and quality of the FISH signals. This is likely due to an increase in the hybridization period that allows more time for probe penetration into the PMCs.

The presence of a background signal in direct labelling was due to the absence of the series of stringent post-detection washes, which are required in the indirect detection protocol. These washes contribute to the elimination of non-specific signals.

Moreover, although studies with *Asparagus* showed some protocol dependence on the genotype, since the same protocol gave different results when it was applied to male plants, diploids, tetraploids and female plants (Guzzo *et al.* 2000), and despite the fact that the arabusta hybrid plants of coffee studied here exhibit different morphologies, and meiotic studies with them have shown that they have different meiotic behaviour with various degrees of abnormalities and fertility (Granato 2010), our successful results with the pre-hydrolysis and FISH procedure were seen uniformly in all of the 17 arabusta hybrid coffee plants that were studied. We have confidence therefore that our technique will open up the possibility of using FISH procedures with pachytene chromosomes in other species and varieties of coffee.

## Conclusions

The FISH protocol for pachytene coffee chromosomes described here opens up the possibility of realizing high-definition physical mapping of these chromosomes by applying different types of probes similar to the repetitive 45S rDNA probe (pTa71) and BACs linked to sequences that have similar characteristics to this probe.

Considering the frequency and intensity of the hybridization signals obtained in the present study, the best results for FISH–rDNA of meiotic coffee chromosomes were obtained for the following: (i) cytological preparations containing PMCs in the early stages of prophase; (ii) slides that were prepared from anthers that were hydrolysed according to Tests B and C for 4 h; (iii) slides that were subjected to 18 h of hybridization; and (iv) the use of a probe with a repetitive sequence that was labelled for indirect detection by an immunochemical reaction.

The protocol established in the present study for coffee chromosomes at the pachytene stage showed the same results for different hybrid plants of arabusta coffee.

## Sources of Funding

This research was partly funded through the Instituto Nacional de Ciência e Tecnologia do Café (INCT-CAFÉ), Brazil (Grant 574009/2008-6) and also by Fundação de Amparo à Pesquisa do Estado de São Paulo (FAPESP), Brazil (Grant 2004/15937-9).

## Contributions by the Authors

C.A.F.P.M. conceived the project, designed the experiments and drafted the manuscript. The experiments were performed by A.A.S.I. and C.A.F.P.M. The manuscript was written by A.A.S.I. and C.A.F.P.M. C.A.F.P.M. edited the final version of the manuscript. Both authors read and approved the final manuscript.

## Conflict of Interest Statement

None declared.
